# Changes in adrenoceptor expression level contribute to the cellular plasticity of glioblastoma cells

**DOI:** 10.1016/j.jphyss.2025.100016

**Published:** 2025-03-28

**Authors:** Yutaro Asaka, Toshio Masumoto, Atsuhito Uneda, Vanessa D. Chin, Yusuke Otani, Tirso Peña, Haruyoshi Katayama, Takuto Itano, Teruhiko Ando, Rongsheng Huang, Atsushi Fujimura

**Affiliations:** a Department of Cellular Physiology, Okayama University Graduate School of Medicine, Dentistry, and Pharmaceutical Sciences, 2–5-1 Shikata-cho, Kita-ku, Okayama 700–8558, Japan; b Division of Health Administration and Promotion, Department of Social Medicine, Faculty of Medicine, Tottori University, 86 Nishi-cho, Yonago, Tottori 683-8503, Japan; c Department of Neurosurgery, Okayama University Graduate School of Medicine, Dentistry, and Pharmaceutical Sciences, 2–5-1 Shikata-cho, Kita-ku, Okayama 700-8558, Japan; d UMass Chan Medical School, UMass Memorial Medical Center, 55 Lake Ave. North, Worcester, MA 01655, USA; e Department of Pathology, Beth Israel Deaconess Medical Center, Harvard Medical School, Boston, MA 02215, USA; f Harvard Medical School, Boston, MA, USA; g Department of Orthopaedic Surgery, Okayama University Graduate School of Medicine, Dentistry, and Pharmaceutical Sciences, 2–5-1 Shikata-cho, Kita-ku, Okayama 700-8558, Japan; h Department of Trauma Orthopedics, The Second Hospital of Dalian Medical University, 467 Zhongshan Rd, Shahekou district, Dalian, Liaoning 116000, China; i Neutron Therapy Research Center, Okayama University, Okayama, Okayama 700–8558, Japan

**Keywords:** Adrenoceptors, Glioma stem-like cells, Differentiated glioma cells, Noradrenaline, Cellular plasticity

## Abstract

Glioblastoma cells are known to regulate their cellular plasticity in response to their surrounding microenvironment, but it is not fully understood what factors contribute to the cells’ changing plasticity. Here, we found that glioblastoma cells alter the expression level of adrenoreceptors depending on their differentiation stage. Catecholamines are abundant in the central nervous system, and we found that noradrenaline, in particular, enhances the stemness of glioblastoma cells and promotes the dedifferentiation potential of already differentiated glioblastoma cells. Antagonist and RNAi experiments revealed that signaling through α1D-adrenoreceptor is important for noradrenaline action on glioblastoma cells. We also found that high α1D-adrenoreceptor expression was associated with poor prognosis in patients with gliomas. These data suggest that glioblastoma cells increase the expression level of their own adrenoreceptors to alter the surrounding tumor microenvironment favorably for survival. We believe that our findings will contribute to the development of new therapeutic strategies for glioblastoma.

## 1 Introduction

Glioblastoma multiforme (GBM) is one of the most frequent malignant brain tumors and still has a poor prognosis despite modern multidisciplinary treatment [38]. The poor prognosis of GBM is thought to be due to the difficulty in excising lesions because of their location in the central nervous system, the aggressive ability of GBM cells to invade surrounding normal brain tissue, and their high resistance to anticancer drugs and radiation [2], [3]. Drastic prognostic improvement has not been achieved since 2005 when a protocol combining temozolomide and radiation therapy was reported to significantly improve progression-free survival in GBM patients [36]. Glioblastoma stem-like cells (GSCs) are known to be the source of these clinical challenges [14]. Although the factors that define a stem cell population vary among cancer types and there is not complete agreement among researchers, four points are generally considered important for GSCs: the ability to self-renew, the ability to maintain an undifferentiated state, the ability to differentiate into multiple lineages, and the ability to form tumors [24], [35]. These properties are thought to be the source of the high degree of heterogeneity in GBM tissues, as well as the source of their resistance to anticancer drugs and radiation [1], [33].

In the field of GBM research, transcriptome results help classify GBM based on molecular biology, which differs from conventional histopathological classification. In 2010, a transcriptome-based method was proposed to classify GBM into four subtypes, "Proneural," "Mesenchymal," "Classical," and "Neural" [42]. As indicated by the name "multiforme," GBMs show a variety of histological features, including areas of marked cell proliferation, necrotic foci, hemorrhagic foci, and pseudopalisading [12], [23]. With the recent development of single-cell RNA sequence technology, it was discovered that GBM tissues are composed of cell populations with different subtype characteristics [11], [32]. That is, in the GBM tissue of one patient sample, it was found that the cell population classified as "proneural subtype" and the cell population classified as "mesenchymal subtype" coexisted [27], [45]. These results are in good agreement with events observed in the Ivy Glioblastoma Atlas Project (Ivy-GAP), a project to link histopathological phenotypes and transcriptome data in GBM tissues [34]. These reports indicate that GBM cells have high plasticity and are adept at reprogramming their cellular properties to adapt to the surrounding environment: GBM cells can easily change their cell properties, which are broadly classified into GSCs and differentiated GBM cells (hereafter referred to as DGCs). Indeed, in the past, four transcription factors have been identified that are important for de-differentiation of DGCs to GSCs [37].

The tumor microenvironment is considered to be an important factor that defines the plasticity of GSCs and DGCs. Tumor cells are continuously influenced by their surrounding environment, and the same is true for GBM tissues, which change their properties by interacting with various components of the tumor tissue [41]. Researched and established factors that constitute the tumor microenvironment include: tumor-associated macrophages [39], [40], gaseous components such as hypoxic environments [21], and liquid components such as trophic factors and metabolites secreted by tissue component cells [26], [4], [46]. In GBM, it has been reported that GSCs and DGCs have a unique mechanism to maintain the other's cellular properties by reciprocally secreting the other’s necessary factors [44]. These reports demonstrate the importance of the interdependent relationship between the tumor microenvironment and GSCs/DGCs as the source of GBM heterogeneity through GSC and DGC plasticity.

In recent years, there have been increasing reports of adrenoceptor-mediated signaling influencing tumor cell characteristics. For example, in prostate cancer, the prognosis is significantly worse in cases where sympathetic and parasympathetic nerve fibers infiltrate the tumor tissue, as these nerve activations promote tumorigenesis and metastasis via the action of adrenergic and muscarinic receptors, respectively [25]. It has also been reported that in breast cancer, stress-induced adrenal medulla-derived adrenaline significantly worsens the prognosis of breast cancer by enhancing the stemness of breast cancer [9]. We previously reported that in malignant peripheral nerve sheath tumor, adrenaline enhanced tumor stemness by activating the YAP/TAZ signal, a core pathway of tumor stem cells [19], [22]. We also reported that in lung cancer, tissue-derived cancer-associated Schwann cells acquire adrenergic synthesis ability, which activates YAP/TAZ signals in surrounding lung cancer cells and enhances cancer stemness [30]. Central nervous system tissues are rich in neurotransmitters with various physiological activities. In addition to major neurotransmitters, such as glutamate and acetylcholine, catecholamines like dopamine and noradrenaline are also used as neurotransmitters in the so-called diffusive modulatory system. Monoamines such as serotonin and histamine are also used as neurotransmitters in the broad projection nervous system. The speculation that these neurotransmitters may influence the properties of GBM has mixed results when tested [5]. For example, it has been reported that changes in dopamine receptor expression confers plasticity on the GBM via transcriptional and metabolic changes [6]. On the other hand, with regard to catecholamines, it was reported in 1972, for example, that noradrenaline inhibits the intracellular uptake of glucose and promotes its degradation in GBM cells in culture [28]. Then in 2021, it was reported that noradrenaline inhibits the invasive potential of GBM cell lines [50]. The effect of catecholamine on GSC/DGC plasticity is not yet well defined. As such, we analyzed the effects of catecholamines on the plasticity of GSCs/DGCs in this study.

## 2 Results

### 2.1 Glioblastoma stem cells have differentiation and dedifferentiation potential

**Fig. 1:**
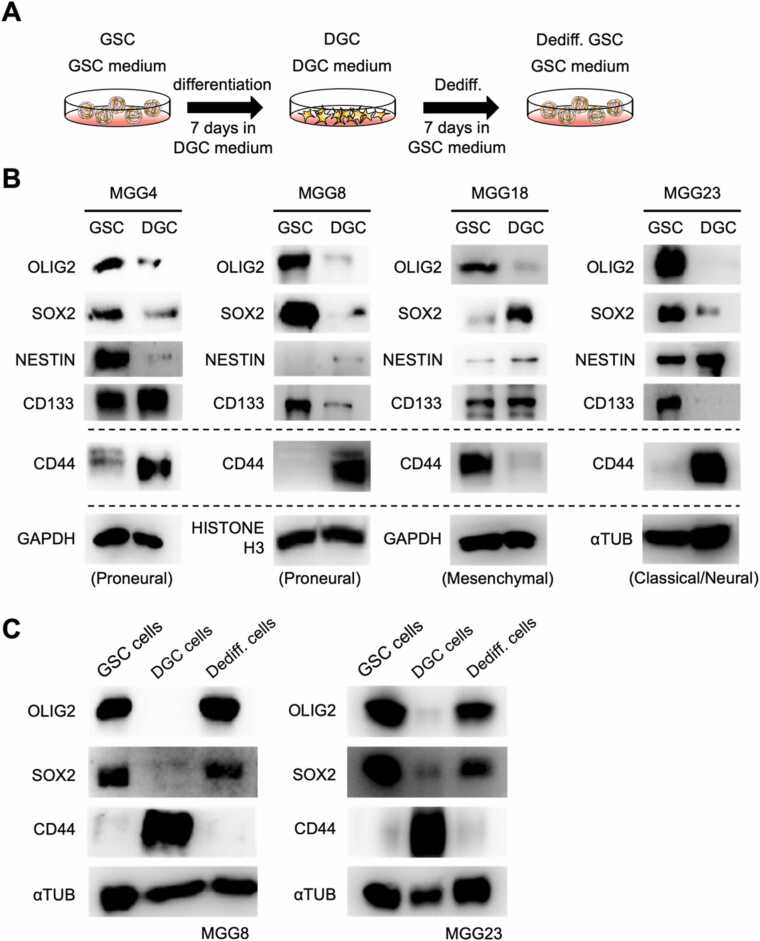
Glioblastoma cells are capable of differentiation and dedifferentiation. (A). Culture schemes for maintenance of GSCs, induction of DGCs, and de-differentiation from DGCs to GSCs. (B). Western blotting results representing transitions in expression levels of undifferentiated and differentiated markers in GSCs and DGCs of human glioblastoma stem cell lines MGG4, MGG8, MGG18 and MGG23. MGG4 and MGG8 cells are classified as proneural subtype. MGG18 and MGG23 cells are classified as mesenchymal and classical/neural subtype, respectively. (C). Western blotting results showing transitions in expression levels of undifferentiated and differentiated markers in MGG8 and MGG23 GSC, DGC, and DGC to GSC de-differentiated cells, respectively.

To test the plasticity of glioblastoma stem cells, we used the glioblastoma stem cell lines MGG4, MGG8, MGG18, and MGG23. All of these cell lines are known to maintain GSC properties when cultured as spheroid in GSC medium. To differentiate these cells, they were trypsinized, suspended in DGC medium, seeded in plates and cultured for 7 days (Fig. 1A). Western blotting analysis of GSC and DGC extract samples of each cell line revealed the expression of the undifferentiated markers NESTIN, OLIG2, SOX2, and CD133 in the GSC samples, while the differentiation marker CD44 was detected in the DGC samples in MGG4, MGG8, and MGG23 (Fig. 1B). As for MGG18 cells, CD44 expression was high and SOX2 expression was low in GSCs, suggesting that it retains the characteristics of the mesenchymal subtype [17]. Furthermore, we confirmed that once the cells were induced to differentiate in DGC differentiation medium, they again acquired expression of undifferentiated markers when cultured back in GSC medium (Fig. 1C). These indicate that the glioblastoma stem cell lines can move between the GSC and DGC states under in vitro culture conditions, suggesting that they possess plasticity at the cellular level.

### 2.2 Glioblastoma stem cells spontaneously differentiate and dedifferentiate in tumor tissue

**Fig. 2:**
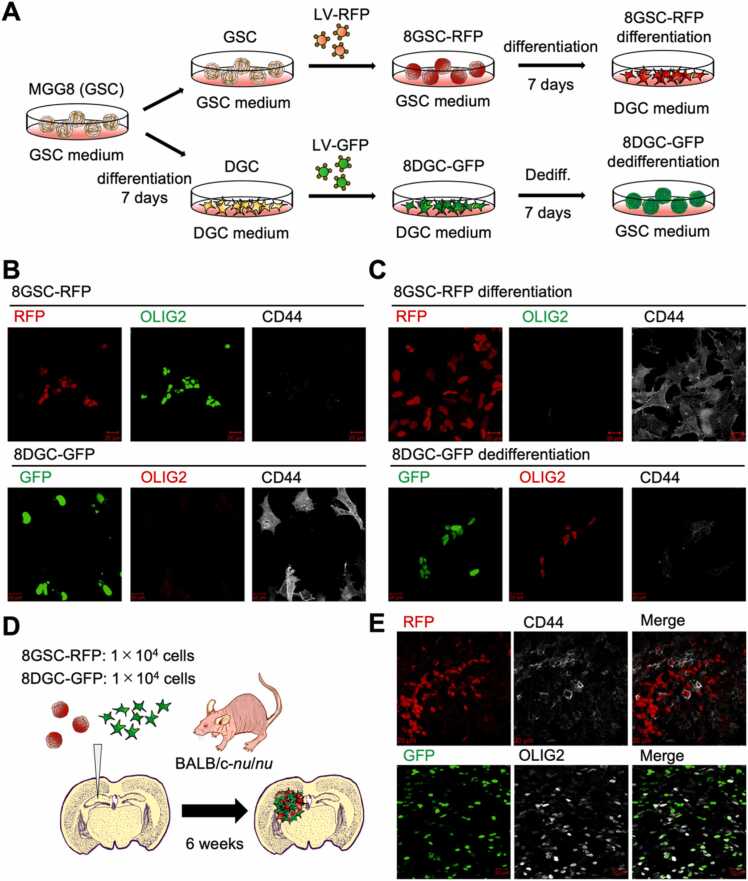
Glioblastoma cells spontaneously differentiate and dedifferentiate in tumor tissue. (A). Scheme representing the method for labeling MGG8; lentivirus LV-GFP or LV-RFP was infected at each stage of GSC or DGC, and GFP and RFP expression was stable before experiments. (B). Immunostaining analysis to confirm undifferentiated and differentiated marker expression in labeled GSCs and DGCs. (C). Immunostaining analysis to evaluate the differentiation and dedifferentiation potential of labeled GSCs and DGCs in vitro*.* (D). Scheme for in vivo evaluation of differentiation and dedifferentiation potential of labeled GSCs and DGCs. (E). Representative immunofluorescent images of the xenograft tissues. Some of the RFP-positive cells (formerly GSCs) express the differentiated marker CD44 and some of the GFP-positive cells (formerly DGCs) express the undifferentiated marker OLIG2. Scale bars are all 20 µm (B, C, D).

We next tested whether similar plasticity could be observed in tumor tissue. This plasticity cannot be demonstrated by immunostaining pathology specimens from GBM patients with undifferentiated or differentiated markers. This is because, as shown in Fig. 1, GBM cells are highly plastic. Even if a cell expresses an undifferentiated marker, it is impossible to assess whether it is a cell that continues to maintain GSC characteristics or if it is a cell that has acquired DGC characteristics then dedifferentiated. The cell labeling and tracking method was employed to demonstrate that both "GSC differentiation to DGC" and "DGC dedifferentiation to GSC" occur in tumor tissues. To generate GBM mouse models using the cell labeling and tracking method, we prepared GSCs and DGCs from MGG8, a cell line that can reproducibly generate orthotopic xenograft GBM models, and lentivirally induced each with red fluorescent protein (8GSC-RFP) and green fluorescent protein (8DGC-GFP), respectively (Fig. 2A). Immunostaining with antibodies against the GSC marker OLIG2 and the DGC marker CD44 confirmed that 8GSC-RFP and 8DGC-GFP maintained the GSC and DGC traits of MGG8, respectively (Fig. 2B). When 8GSC-RFP was cultured in DGC medium for 7 days, the expression of OLIG2 disappeared and CD44 was expressed (Fig. 2C, top). When 8DGC-GFP was cultured in GSC medium for 7 days, we confirmed that OLIG2 was expressed and CD44 was lost (Fig. 2C, bottom). Since we confirmed that both 8GSC-RFP and 8DGC-GFP retain plasticity in vitro, we next generated an orthotopic xenograft GBM model. We transplanted 1x 10^4^ each of 8GSC-RFP and 8DGC-GFP in the left brain parenchyma of *BALB/c-nu/nu* mice (Fig. 2D) using a stereotaxic instrument. Six weeks after cell transplantation, frozen sections of perfusion-fixed cerebral tissue were prepared and immunostained with OLIG2 and CD44. The results showed that some of RFP-positive cells expressed the differentiation marker CD44 (Fig. 2E, top) and some of GFP-positive cells expressed the undifferentiation marker OLIG2 (Fig. 2E, bottom). These data indicate that GBM cells can move between undifferentiated and differentiated states in tumor tissue.

### 2.3 Glioblastoma cells express varying levels of adrenergic receptors depending on their level of differentiation

**Fig. 3:**
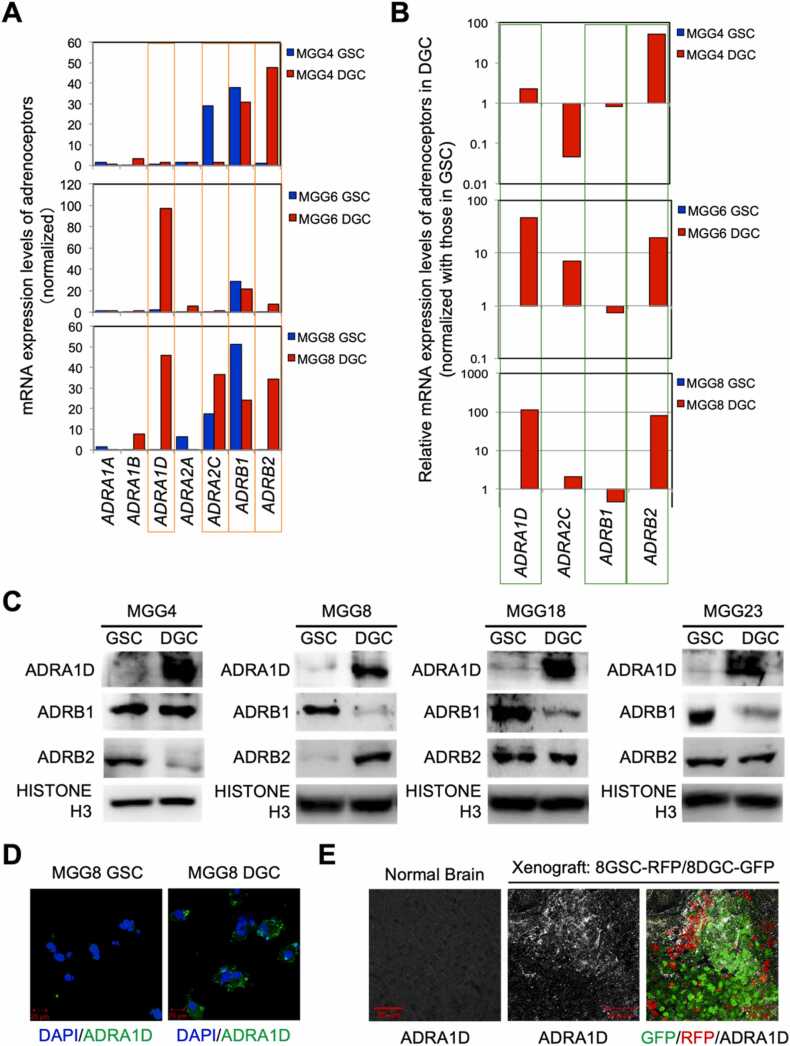
Glioblastomas alter their adrenoceptor expression levels depending on their stage of differentiation. (A). Number of RNA sequence reads representing the expression of mRNAs encoding adrenoceptors in GSCs and DGCs of MGG4, MGG6, and MGG8. *ADRA1D*, *ADRA2C*, *ADRB1*, and *ADRB2*, which are well expressed and particularly variable, are highlighted with orange lines. (B). Graph showing the relative expression levels of *ADRA1D*, *ADRA2C*, *ADRB1*, and *ADRB2* in GSCs and DGCs of MGG4, MGG6, and MGG8 as a ratio of DGCs to GSCs. The vertical axis is shown in logarithm; of the four genes, those representing a common pattern of variation in the three cell lines are highlighted with green lines. (C). Western blotting results evaluating protein expression of ADRA1D, ADRB1, and ADRB2 in GSCs and DGCs of MGG4, MGG8, MGG18, and MGG23. (D). Immunostaining images showing changes in ADRA1D expression in GSCs and DGCs of MGG8. (E). Immunostaining image showing ADRA1D expression in tumor tissue of orthotopic xenografts composed of 8GSC-RFP and 8DGC-GFP. ADRA1D, which is upregulated in the differentiated state, is positive in some RFP-positive cells (formerly GSC cells) and negative in some GFP-positive cells (formerly DGC cells), indicating that, like undifferentiated and differentiated markers, the expression pattern of ADRA1D is also heterogeneous. Staining for ADRA1D in normal mouse brain tissue is shown as a control.

GBM cells are known to dramatically alter their transcriptome during the differentiation process from GSCs to DGCs. We speculated that there could be differences in the expression levels of catecholamine receptors between GSCs and DGCs. We further speculated that if differences in these expression levels occur, it could be supported by a combination of the following: catecholamine receptor expression influences the effect of catecholamines on cells, central nervous system tissue is catecholamine-rich, and adrenoceptor signaling has been reported to be important in regulating the activity of YAP/TAZ, a core factor in the regulation of GSC/DGC plasticity [7]. As such, we hypothesized that changes in adrenoceptor expression levels may be involved in the regulation of GSC/DGC plasticity. To verify this, we analyzed the public dataset and identified changes in adrenoceptor expression levels in GSC/DGC. We analyzed the transcriptome data of GSCs and DGCs in three cell lines, including MGG4, MGG6, and MGG8, and found that the expression levels of adrenoceptors could change (Fig. 3A). The mRNA expression levels of *ADRA1A*, *ADRA1B*, and *ADRA2A* gene in GSCs and DGCs cell lines were low in both cases (Fig. 3A). Among the remaining *ADRA1D*, *ADRA2C*, *ADRB1,* and *ADRB2* genes, we focused on the *ADRA1D*, *ADRB1*, and *ADRB2* genes that showed a similar variation pattern among the cell lines (Fig. 3B). Although the range of variation varied slightly among cell lines, the expression levels of the *ADRA1D* and *ADRB2* genes were found to increase in DGCs, while the expression level of the *ADRB1* gene was maintained at a high level in DGCs and GSCs. To determine whether these events were also true at the protein level, we performed western blotting analysis of GSC and DGC samples from the glioblastoma stem cell lines MGG4, MGG8, MGG18, and MGG23, respectively. In all cell lines, the α_1D_ adrenergic receptor protein levels were abundant in DGCs (Fig. 3C). α_1_D adrenoceptors tended to be higher in DGC and β_1_ adrenoceptor transcript levels tended to be higher in GSCs, while changes in β_2_ adrenoceptor expression varied between cell lines, indicating a divergence between transcript and protein level variation. Using immunostaining, only changes in the expression level of the α_1D_ adrenoceptor could be confirmed by this method (Fig. 3D). Furthermore, in tumor tissues of the orthotopic xenograft GBM model with 8GSC-RFP and 8DGC-GFP using the cell labeling and tracking method, immunostaining verified that RFP-positive cells (cells that were once maintained in an undifferentiated state as GSCs cells) abundantly express the α_1D_ adrenergic receptor, which is upregulated in the differentiated state (Fig. 3E). These data demonstrate that glioblastoma cells change their mode of adrenergic receptor expression depending on their level of differentiation.

### 2.4 Glioblastoma stem cells enhance self-renewal capacity in response to noradrenergic stimulation

**Fig. 4:**
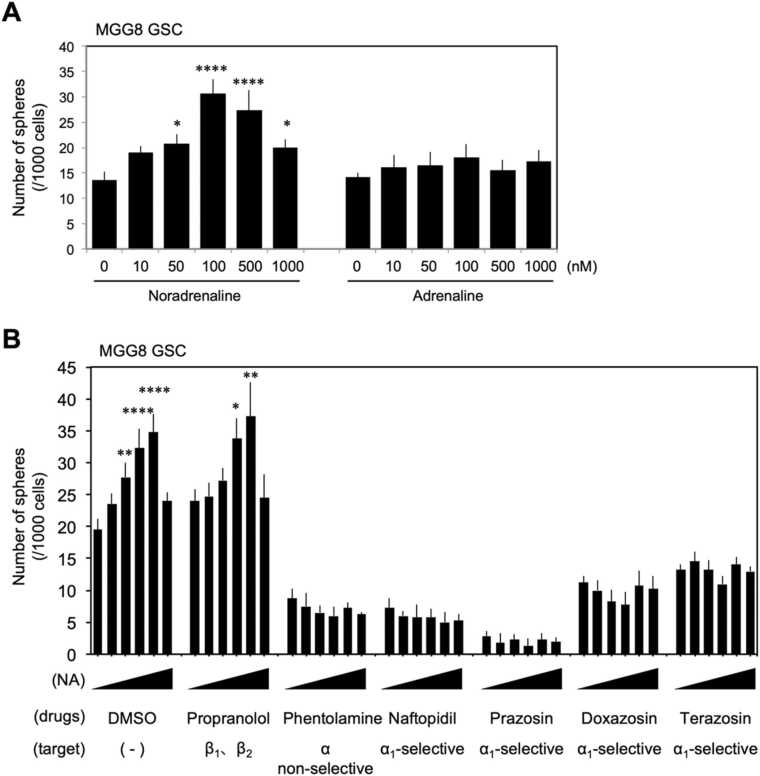
Glioblastoma stem cells enhance their self-renewal capacity in response to noradrenergic stimulation. (A). Evaluation of sphere forming capacity in a scaffold-independent culture environment. Number of spheres formed per 1000 were counted. n = 4. (B). Sphere formation assay to assess whether the concentration-dependent self-renewal enhancing effects of noradrenaline are inhibited by antagonists of the respective adrenergic receptors. The same dilution sequence as in (A) was employed for noradrenaline concentrations. n = 4. * : p < 0.05, * *: p < 0.01, * ** : p < 0.001, * ** *: p < 0.0001.

Because glioblastoma cells showed variable levels of adrenergic receptor expression depending on the differentiation level of GSCs and DGCs and adrenergic signaling can modify the stemness [19], we speculated that the effects of catecholamines on glioblastoma cells differ between the differentiation states of GSCs and DGCs and the stemness changes according to the adrenaline signaling cascade. To test this idea, we analyzed the effects of noradrenaline and adrenaline in the differentiation states of GSCs and DGCs, respectively. The stemness of glioblastoma stem cells can be qualitatively and quantitatively assessed by analyzing the efficiency of sphere formation in serum-free medium in a scaffold-independent culture environment as self-renewal capacity. The human glioblastoma stem cell line MGG8 was isolated into single cells, resuspended in GSC medium at 1000 cells/1.5 mL, and seeded into 24-well flat-bottom ultra-low-adhesion surface plates with scaffold-independent environment at 1.5 mL. To test the effects of noradrenaline and adrenaline on self-renewal capacity, final concentrations of 0, 10, 50, 100, 500, and 1000 nM were added, respectively. Interestingly, noradrenaline significantly enhanced self-renewal capacity in a concentration-dependent manner up to 100 nM, whereas no significant self-renewal enhancing effect was observed at the adrenaline concentration we tested (Fig. 4A). Noradrenaline is considered to have good agonist activity at the α_1_ adrenergic receptor, α_2_ adrenergic receptor, and β_1_ adrenergic receptor, but very weak at the β_2_ adrenergic receptor. On the other hand, adrenaline is generally considered to be a good agonist for both receptors, although it has high and low titers for each receptor. The results that noradrenaline but not adrenaline enhanced the self-renewal capacity of MGG8 GSCs suggests that the signal input from the β_2_ adrenergic receptor is not significantly involved in the regulation of GSC self-renewal ability. In order to test which of the receptors is important for the enhancement of GSC self-renewal capacity, we next evaluated the receptor contribution on the sphere formation at graded noradrenergic concentrations similar to Fig. 4A using each antagonist. First, we used propranolol, a nonselective β-adrenoceptor antagonist, to verify the involvement of β_1_ adrenergic receptors and found that 10 μM of propranolol treatment had no effect on the potentiation of self-renewal induced by noradrenaline (Fig. 4B). Next, we evaluated the non-selective α-adrenergic receptor antagonist phentolamine at 10 μM, and we found that phentolamine significantly suppressed both the self-renewal capacity and the noradrenaline-induced enhancement of self-renewal capacity of GSCs (Fig. 4B). Furthermore, α_1_ adrenoceptor-selective antagonists naftopidil, prazosin, doxazosin, and terazosin significantly suppressed the self-renewal ability of GSCs, suppressing the noradrenaline-induced self-renewal potentiation (Fig. 4B). In particular, the inhibitory effect of prazosin was strong (Fig. 4B), and it was found that prazosin alone exerted a very strong inhibitory effect. These data indicate that signaling through α_1_ adrenoceptors is important for the self-renewal enhancing effect of noradrenaline on glioblastoma stem cells.

### 2.5 DGC enhances de-differentiation potential by noradrenergic stimulation

**Fig. 5:**
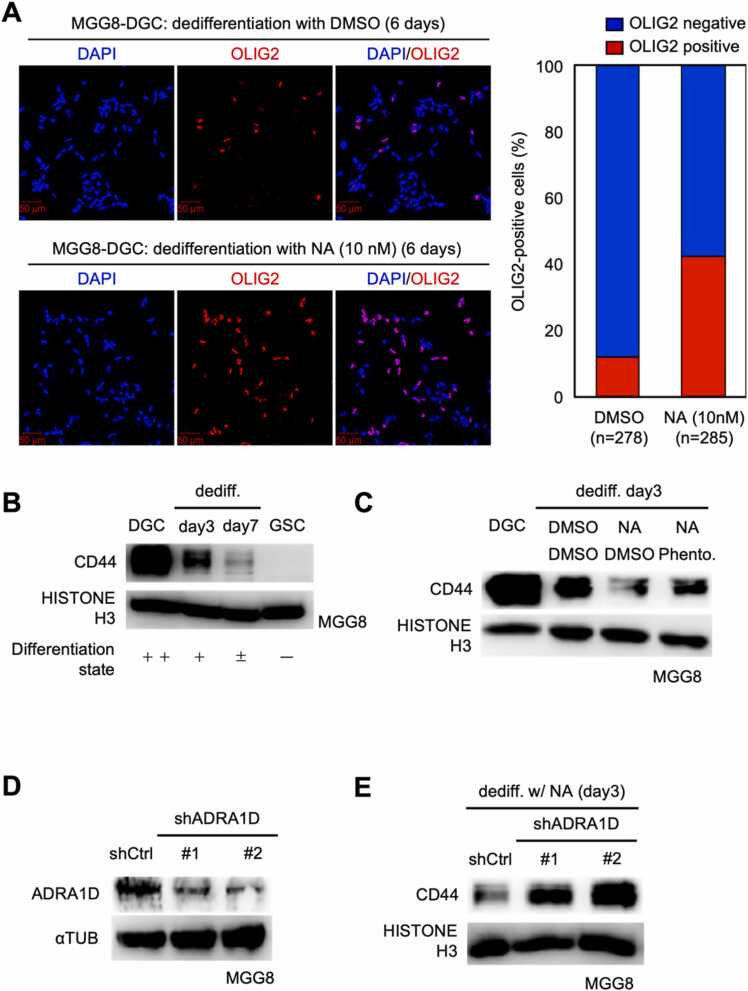
Noradrenergic stimulation of differentiated glioblastoma cells enhances their dedifferentiation potential. (A). MGG8 DGCs were subjected to induction of dedifferentiation in GSC medium containing DMSO or Noradrenaline (10 nM) for 6 days and stained with OLIG2, an undifferentiated marker. The graph on the right represents the percentage of OLIG2-positive cells among all cells. Scale bar is 50 µm. (B). MGG8 DGCs were subjected to dedifferentiation induction for 3 days (day3) or 7 days (day7) in GSC medium and the expression of the differentiation marker CD44 was analyzed by western blotting. Qualitative differentiation status is presented as a summary. (C). DGCs of MGG8 were subjected to dedifferentiation induction for 3 days in GSC medium containing DMSO or noradrenaline (10 nM) and phentolamine (10 μM), and the expression of the differentiation marker CD44 was analyzed. (D). Knockdown by shRNA was confirmed by western blotting. (E). DGCs of MGG8 prepared as in (D) were subjected to dedifferentiation induction for 3 days in GSC medium containing noradrenaline (10 nM) and the expression of the differentiation marker CD44 was analyzed.

Previous data have shown that noradrenaline has a significant impact on glioblastoma stemness through signaling via the α_1_ adrenoceptor (Fig. 3, Fig. 4). Since the molecular mechanisms that maintain stemness and those that acquire and reactivate stemness are similar [37], we hypothesized that noradrenergic stimulation may promote the process of dedifferentiation from DGCs to GSCs. To test this hypothesis, MGG8 GSCs were cultured in DGC medium for 7 days to generate MGG8 DGCs and then induced dedifferentiation by changing the medium to GSC medium. As shown in Fig. 5A, immunostaining analysis of MGG8 DGCs cultured for 6 days in GSC medium containing vehicle or 10 nM noradrenaline showed that the number of cells positive for the undifferentiated marker OLIG2 was increased in the samples treated with noradrenaline. Western blotting analysis showed that the expression of CD44, a differentiation marker, was attenuated 3 days after induction of dedifferentiation and further attenuated 7 days later (Fig. 5B). Furthermore, the expression of CD44 was significantly weakened in the noradrenaline-treated samples compared to the negative control samples (Fig. 5C). This suppression of CD44 expression could be inhibited by the addition of 10 μM phentolamine, suggesting that the dedifferentiation-promoting effect of noradrenaline on MGG8 DGCs is mediated by the α-adrenergic receptor.

As verified in Fig. 3, the α_1D_ adrenergic receptor is commonly upregulated in DGCs of all glioblastoma stem cell lines. We therefore tested whether the α_1D_ adrenoceptor is involved in augmenting the noradrenergic-induced dedifferentiation of DGCs. MGG8 DGCs that had completed differentiation induction were transfected with lentivirus expressing short hairpin RNA (shRNA) against the *ADRA1D* gene (encoding the α_1D_-adrenoceptor) (Fig. 5D) and subjected them to dedifferentiation in GSC medium. We found that CD44 attenuation was suppressed in shRNA-*ADRA1D*-induced cells compared to the shRNA-negative control (Fig. 5E). These data indicate that noradrenaline stimulation of α_1D_ adrenoceptors enhances GSC/DGC plasticity.

### 2.6 α_1D_ adrenoceptor expression levels correlate with glioblastoma prognosis

**Fig. 6:**
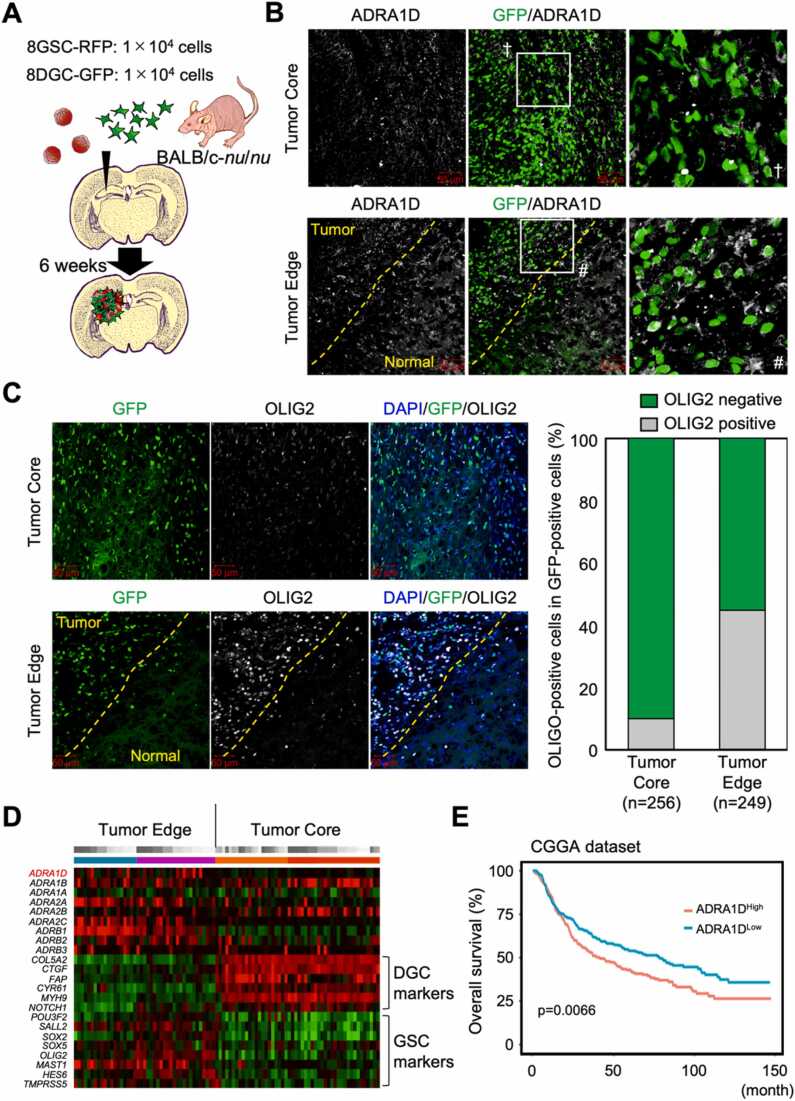
α_1D_ adrenoceptor expression levels correlate with glioblastoma prognosis. (A). Scheme for in vivo evaluation of changes in the expression pattern of α_1D_ adrenoceptors in labeled GSCs and DGCs. (B). Immunostaining images representing the altered expression pattern of α_1D_ adrenoceptors in vivo in labeled GSCs and DGCs. Tumor core (upper panels) and tumor edge (lower panels) are shown, respectively. The yellow dashed line represents the region that appears to be the boundary between the tumor edge and normal brain tissue. The stained images with the white squares strongly magnified are placed in the rightmost panel, respectively. (C). Immunostaining images representing the altered expression pattern of OLIG2 in vivo in labeled GSCs and DGCs. Tumor core (upper panels) and tumor edge (lower panels) are shown, respectively. The right graph shows the percentage of OLIG2-positive cells among all GFP-positive cells, at the tumor core and at the tumor edge, respectively. (D). Each adrenoceptor gene, differentiation marker gene, and undifferentiated marker gene was analyzed by Ivy-GAP. (E). Brain tumor patients deposited in the CGGA dataset were grouped into *ADRA1D* high and low expression and their survival curves were plotted.

Glioblastoma tissues are known to be highly heterogeneous and contain a mixture of cells at various stages of differentiation within the tissue. To examine the regions where α_1D_ adrenergic receptors are expressed in glioblastoma tissues, we created an orthotopic xenograft GBM model with 8GSC-RFP and 8DGC-GFP (Fig. 6 A). We performed immunostaining with antibodies against the α_1D_ adrenoceptor, and found that 8DGC-GFP localized at the invasion tip expressed the α_1D_ adrenoceptor, while the tumor center did not (Fig. 6B). Interestingly, OLIG2-positive cells in 8DGC-GFP localized at the invasive tip were found to be more abundant than those at the tumor center (Fig. 6 C). This complements the Ivy-GFP data, which shows higher expression of *ADRA1D* gene and undifferentiated-related markers at the invasive tip and higher expression of differentiation-related markers at the tumor center (Fig. 6D). These data indicate that the tumor is efficiently dedifferentiated at the invasive tip by making it more susceptible to the action of noradrenaline derived from the surrounding normal brain tissue.

Finally, to examine whether α_1D_ adrenergic receptors can actually influence the prognosis of brain tumors, we analyzed the association between the expression level of α_1D_ adrenergic receptors and overall survival of brain tumors, based on data deposited in the Public database. We sourced in the Gliovis dataset and we found that in the Chinese Glioma Genome Atlas (CGGA), the prognosis of patients with high *ADRA1D* gene expression was significantly worse than that of patients with low expression (Fig. 6E, p value=0.0066). These data suggest that the expression level of the α_1D_ adrenoceptor defines the prognosis of brain tumors, supporting our previous experimental results.

## 3 Discussion

### 3.1 Role of neurotransmitter receptors expressed by glioblastoma cells in glioblastoma progression

As we have shown in this study, glioblastomas modulate the expression level of adrenoceptors according to their level of differentiation (Fig. 3). Both of these ligands, noradrenaline and adrenaline, are neurotransmitters that are abundant in CNS tissues, and it is thought that glioblastomas alter their environment to be favorable for their own survival via expression of their own adrenoceptors. Central nervous tissue is rich in catecholamines compared to other body tissues. For example, the concentration of noradrenaline in rodent brain parenchyma has been calculated to be generally in the range of 10^1^∼10^3^ nM, although there is considerable range due to the varying methods of detection and sites analyzed in different reports [18], [29]. Considering that the detected noradrenaline concentration in human blood is 10^-1^∼10^0^ nM [10] and that the estimated calculated noradrenaline concentration between the human sympathetic nervous system and its effector is 10^0^∼10^1^ nM [15], the noradrenaline concentration in central nervous tissue is about 10 times higher than in other body tissues. In in vitro experiments, the self-renewal enhancing effect of noradrenaline was strongest at 100 nM (Fig. 4A). Given that noradrenaline levels in the brain parenchyma are generally 10^1^∼10^3^ nM, the brain parenchyma is considered to be a favorable environment for the maintenance and enhancement of self-renewal of GSCs. In addition, as shown in Fig. 5 and 6, α_1D_ adrenoceptor signaling by noradrenaline enhances the dedifferentiation ability of DGCs, suggesting that glioblastomas are complying with their own tumor microenvironment by utilizing noradrenaline present in normal brain tissue to dedifferentiate DGCs to GSCs.

In this study, we focused on noradrenaline, which was thought to have a significant effect on both GSCs and DGCs, but it would be worthwhile to investigate the involvement of other neurotransmitters in the future. For example, although adrenaline had no significant effect on the maintenance of self-renewal capacity of GSCs, the expression of β_2_ adrenoceptors, which are activated by adrenaline, was found to be significantly enhanced in DGCs of some glioblastoma cells. It is possible that β_2_ adrenergic stimulation via adrenaline could have some effect on the plasticity and other properties of DGCs, considering previous reports that adrenaline enhances the stemness in many cancer types [19], [9]. We have also found that the expression of CHRM2, a muscarinic acetylcholine receptor, is markedly enhanced by induction of differentiation (unpublished data). It is reasonable to expect that acetylcholine, which is important for neurotransmission in the central nervous system, in addition to monoamines, could affect glioblastoma plasticity.

### 3.2 Treatment strategy for glioblastoma based on the expression pattern of adrenergic receptors

We have found that the nonselective α adrenoceptor antagonist phentolamine and the α_1D_ adrenoceptor-selective antagonists naftopidil, prazosin, doxazosin, and terazosin inhibit glioblastoma stem cell plasticity. All of these drugs are used in daily medical practice. α_1D_ adrenoceptor selective antagonists are widely prescribed to treat hypertension and benign prostatic hyperplasia [43]. In addition, three drugs, prazosin, doxazosin, and terazosin, are considered to be drugs that can cross the blood-brain barrier, albeit not actively based on analysis of ADMET, (absorption, distribution, metabolism, excretion, and toxicity). Clinical studies are underway to determine whether these drugs can actually be used as a treatment for post-traumatic stress disorder (also known as PTSD) [43]. Thus, compounds that can cross the blood-brain barrier and inhibit the molecular mechanism newly discovered by us in this study are expected to inhibit the progression of glioblastoma by limiting its plasticity. Further validation of the drug in an orthotopic xenograft mouse model should be conducted to determine whether or not the drug is clinically investigational. Alternatively, a retrospective observational study could be conducted to determine whether there is a difference in overall survival between glioblastoma patients who were taking an α_1D_ adrenoceptor selective antagonist compared to those who were not.

### 3.3 Limitation of this study and future issues

In this study, we demonstrated that noradrenergic signaling via α_1D_ adrenoceptor regulates glioblastoma stem cell plasticity and that α_1D_ adrenoceptor can influence glioblastoma progression. However, the involvement of other adrenoceptors in GSC/DGC plasticity has not been fully investigated. For example, in GSCs, the expression of α_2_ adrenoceptors is higher than that of α_1_ adrenoceptors, but could these receptors be involved in the regulation of glioblastoma plasticity and stemness? Or, could the β adrenoceptors be involved in glioblastoma progression? Further research is needed to answer these questions. We hope that this study will be the first step toward an integrated understanding of adrenergic receptor signaling in glioblastoma cells.

Adrenoceptors are GPCR protein, and downstream signaling pathways are activated when noradrenaline and adrenaline bind the receptors. For example, the stimulation of α adrenoceptors by noradrenaline, which we focused on in this study, activates phospholipase C through the action of G_q_ proteins. Phospholipase C causes an increase in intracellular calcium via inositol triphosphate and diacylglycerol, and ultimately activates protein kinase C (PKC). It is also believed that when the β-adrenoceptor is activated, the G_s_ protein activates adenylate cyclase, which raises cyclic AMP and ultimately activates protein kinase A (PKA). PKC and PKA have been extensively studied as factors that determine the properties of many tumor cells.

Recently, YAP/TAZ, the transducers of the Hippo signaling pathway, were identified as the master factor for GSC/DGC plasticity [7]. YAP/TAZ are thought to be essential for maintaining cancer stem cells in various cancer types [48], [8], not only GBM. Castellan et al. reported that YAP/TAZ activation is essential in both types of GBM during the process of dedifferentiation from DGC to GSC [7]. This report represents the importance of the plasticity control mechanism of GSCs/DGCs via YAP/TAZ signaling as the source of heterogeneity in GBM tissues. Central nervous system tissues are composed of soft extracellular matrix, which is very unfavorable for YAP/TAZ signaling, which regulates GSC/DGC plasticity. It is widely recognized that YAP/TAZ signaling is highly regulated by mechanotransduction and is most active when it is on a rigid extracellular matrix [49]. In the "soft" brain, how can YAP/TAZ activity be sufficient to impose GSC/DGC plasticity? Interestingly, YAP/TAZ is reported to be activated by PKC and inhibited by PKA [16], [47]. Based on the molecular mechanism discovered by us in this study, although glioblastomas have an unfavorable soft environment for YAP/TAZ activation, they may ensure plasticity by exploiting the surrounding noradrenaline to activate PKC and maintain YAP/TAZ activity. Further analysis of molecular mechanisms focusing on YAP/TAZ is necessary to advance our integrated understanding of adrenergic receptor signaling in glioblastoma cells.

## 4 Materials and methods

### 4.1 Cell cultures

The human GBM stem cell lines MGG4, MGG8, MGG18, and MGG23 were provided by Dr. Hiroaki Wakimoto (Brain Tumor Research Center, Massachusetts General Hospital). These cells were grown in Neurobasal medium (Thermo Fisher Scientific) with 1x B-27 supplement (Thermo Fisher Scientific), 1x N-2 supplement (Thermo Fisher Scientific), 20 ng/ mL human epidermal growth factor (h-EGF) (Fujifilm-Wako), 20 ng/mL human basic fibroblast growth factor (h-bFGF) (Fujifilm-Wako), 10 μg/mL heparin sodium (Fujifilm-Wako), and 1x penicillin-streptomycin-L-glutamine solution (Fujifilm-Wako) (hereinafter referred to as GSC medium) in a humid chamber at 37 ℃, 5 % CO_2_.

For differentiation of MGG4, MGG8, MGG18, and MGG23, cells were grown in Dulbecco's Modified Eagle Medium (DMEM) (Fujifilm-Wako), 10 % Fetal bovine serum (FBS) (Corning), 50 ng/mL human bone morphogenetic protein 2 (h-BMP-2) (Fujifilm-Wako), and 1x penicillin-streptomycin-L-glutamine solution (hereinafter referred to as DGC medium). MGG cells isolated into single cells were resuspended in DGC medium and then placed on Matrigel Growth Factor Reduced (GFR) Basement Membrane Matrix (Corning)-coated 6 well plates. The DGC medium was changed every 3 days.

### 4.2 Sphere-formation assay

The sphere formation assay was performed as previously described [20]. Briefly, MGG cells separated into single cells were resuspended in GSC medium at 1000 cells/1.5 mL and seeded into 24-well flat bottom ultra-low attachment plates (Corning). At least 4 well replicas per condition were set up to allow for statistical tests. Catecholamines and drugs were dissolved in DMSO; Adrenaline, Noradrenaline, Propranolol, and Phentolamie were purchased from Tokyo Kasei Kogyo Co. (catalog numbers: A0173, A0906, P0995, P1985). Naftopidil, Prazosin, Doxazosin, and Terazosin were purchased from Selleck Biotech Corporation (catalog numbers: S2126, S1424, S1324, S2059).

### 4.3 Lentiviruses

To perform the cell labeling and tracking method, LV-GFP (Addgene, catalog no. 25999) and LV-RFP (Addgene, catalog no. 26001) were employed as backbone plasmids for lentiviruses, respectively. In addition, to create lentiviruses expressing shRNAs, pLKO.1 puro shRNA-scramble (Addgene, catalog #1864) and pLKO.1 puro shRNA-human ADRA1D #1 and pLKO.1 puro shRNA-human ADRA1D #2 were employed as backbone plasmids. To create virus particles, 10 μg of the above backbone plasmids, 7.5 μg of the psPAX2 plasmid (Addgene, catalog no. 12260), and 2.5 μg of the pMD2. G plasmid (Addgene, catalog no. 12259), for a total of 20 μg, were added to 60 μL of TransIT- LT1 Transfection Reagent (Takara Bio) and 1.5 mL of Opti-MEM Reduced Serum Medium, no phenol red (Thermo Fisher Scientific) and then transfected into HEK293FT cells (Thermo Fisher Scientific) cultured at 80 % confluency on 10 cm culture dishes. After 16 h of transfection, the medium was replaced with GSC medium and cultured for another 48 h. The collected culture supernatant was passed through a polysulfone syringe filter (pore size 0.45 µm) (KURABO) as the virus-containing medium. For infection, the cells were cultured for 72 h using a mixture of the medium and virus-containing medium at a volume ratio of 3:1. The recombinant DNA experiments in this study were conducted with the approval of the committee of Okayama University (Approval No. 17138).

### 4.4 Orthotopic xenograft GBM model mice

To create orthotopic xenograft GBM model mice, 8GSC-RFP and 8DGC-GFP were created by introducing LV-RFP and LV-GFP into GSC and DGC of MGG8, respectively. The left brain parenchyma (1 mm anteriorly and 2 mm laterally from Bregma, and at a depth of 3 mm) of BALB/cSlc-*nu/nu* (hereafter referred to as *BALB/c-nu/nu* mice) fully anesthetized with a triad of medetomidine (0.3 mg/kg), midazolam (4 mg/kg) and butorphanol (5 mg/kg) were inoculated with 1x 10^4^ cells each of 8GSC-RFP and 8DGC-GFP using a stereotaxic fixation device. Six weeks after inoculation, frozen sections of cerebral tissue perfusion-fixed with 4 % paraformaldehyde (4 % PFA) (Fujifilm-Wako) under anesthesia were prepared and used for immunostaining experiments. Animal experiments in this study were conducted after approval by the Okayama University Animal Experiment Committee (Approval No. OKU-2021331).

### 4.5 Western blotting analysis

Samples for western blotting were prepared as previously described [13]. Briefly, cells were dissolved in cell lysis buffer (20 mM Tris-HCl (pH=7.5), 150 mM NaCl, 1 mM EGTA, 1 mM EDTA, 1 % Triton X-100, 1 ×cOmplete EDTA-free Protease Inhibitor Cocktail, 1 ×PhosSTOP), lysed using a sonicator. After centrifugation, the supernatant of cell lysate were collected and mixed with sample buffer (250 mM Tris-HCl (pH 6.8), 10 % Sodium Dodecyl Sulfate, 0.008 % Bromophenol Blue, 40 % Glycerol, 20 % 2-Mercaptoethanol) and incubated at 95 ℃ for 5 min. Proteins were separated by SDS-PAGE and then transferred to PVDF membrane and blocked with 0.5 % skim milk dissolved in 1x TBS-T for 1 h at room temperature. After blocking, the membrane was incubated with the primary antibody in 0.1 % skim milk at 4 ℃ for 16 h. After 16 h, the membrane was washed three times with 1x TBS-T for 5 min, and then incubated with the secondary antibody in 0.1 % skim milk for 1 h at room temperature. 1 h later, the membrane was washed three times with 1x TBS-T for 5 min, and then incubated with HRP chemiluminescent substrate solution (Bio-Rad). The signals were detected using ChemiDoc Touch Imaging System (Bio-Rad). The antibodies used for Western blotting are listed in the table below (Table 1).

**Table 1:** The antibodies used for Western blotting.

**Antibodies**	**Dilution**	**Manufacturer**	**Catalog #**
HistonH3	1:10000	Proteintech	17168 −1-AP
Alpha tubline mouse McAb	1:10000	Proteintech	66031 −1 −1 g
GAPDH mouse McAb	1:10000	Proteintech	60004 −1 −1 g
CD133 rabbit polyclonal	1:2000	Proteintech	18470 −1-AP
Sox−2 (Y−17) Goat polyclonal IgG	1:2000	Santa Cruz	sc−17320
Rb mAb to OLIG2	1:2000	abcam	ab109186
Rb pAb to CD44	1:2000	abcam	ab157107
Anti-Nestin antibody produced in rabitt	1:3000	Sigma Aldrich	N5413
β₁-AR(V−19) rabbit polyclonal IgG	1:2000	Santa Cruz	sc−568
α₁D-AR (R20) goat polyclonal IgG	1:2000	Santa Cruz	sc−1475
ADRB2 Rabbit Poly Ab	1:2000	Proteintech	13096 −1-AP
Anti-mouse IgG HRP-linked antibody	1:2000	Cell Signaling Technology	7076P2
Anti-rabbit IgG HRP-linked antibody	1:2000	Cell Signaling Technology	7074S
Anti-Goat IgG-Peroxidase antibody	1:2000	Sigma Aldrich	A5420

### 4.6 Immunofluorescent analysis

Immunofluorescent analyses were performed as previously described [31]. Cells for immunofluorescent analysis were seeded on chamber slides coated with Matrigel Growth Factor Reduced (GFR) Basement Membrane Matrix. After removal of medium, cells were fixed with 4 % PFA for 15 min at room temperature, and blocked with blocking reagent (K.A.C. Co., Ltd.) for 1 h at room temperature. After the blocking solution was removed, the primary antibody diluted with blocking reagent was added to the samples and allowed to react for 16 h at 4 ℃. After 16 h, the samples were washed three times with PBS for 5 min, and the secondary antibody diluted with blocking reagent at an appropriate ratio was added to the samples. After 1 h, the samples were washed three times with PBS for 5 min, and then embedded with DAPI Fluoromount-G (Southern Biotech Co., Ltd.).

For immunofluorescent staining of the specimens from the orthotopic xenograft glioblastoma model, perfusion-fixed mouse brain tissue was embedded in OCT compound (Sakura Finetek Japan) and frozen sections 10 µm thick were prepared in a cryostat (LEICA). Then, the sections were attached to glass slides (Matsunami glass), air-dried, washed with PBS, and blocked with blocking reagent for 1 h at room temperature. The subsequent steps are the same as for cell staining. A confocal microscope system (LSM780, ZEISS Corporation) was used for observation and image acquisition. The antibodies used for immunofluorescence analysis are listed in the table below (Table 2).

**Table 2:** The antibodies used for immunofluorescence analysis.

**Antibody**	**Dilution**	**Manufacturer**	**Catalog #**
anti mouse/human CD44	1:200	BioLegend	103002
Rb mAb to OLIG2	1:200	abcam	ab109186
α₁D-AR (R20) goat polyclonal IgG	1:200	Santa Cruz	sc−1475
Alexa Fluor™ Plus 647, Donkey anti-Rat IgG (H+L)	1:400	Invitrogen	A48272
Alexa Fluor™ Plus 647, Donkey anti-Rabbit IgG (H+L)	1:400	Invitrogen	A32795
Alexa Fluor™ Plus 647, Donkey anti-Goat IgG (H+L)	1:400	Invitrogen	A32849
Alexa Fluor™ Plus 488, Donkey anti-Goat IgG (H+L)	1:400	Invitrogen	A32814

### 4.7 Analysis of public dataset

The Ivy-Glioblastoma Atlas Project (Ivy-GAP) was used to validate the site-specific gene expression patterns of glioblastomas (https://glioblastoma.alleninstitute.org). We also analyzed the CGGA dataset using the GlioVis data portal to assess the correlation between *ADRA1D* gene expression levels and prognosis in brain tumor patients (http://gliovis.bioinfo.cnio.es).

### 4.8 Statistics

Unpaired two-tailed *t*-test was used to evaluate the difference between the two groups. One-way ANOVA (analysis of variance) followed by Turkey-Kramer method was used to compare two or more groups. p < 0.05 was considered statistically significant. p < 0.05, p < 0.01, p < 0.001, and p < 0.0001 were denoted by * , * *, * ** *, and * ** *, respectively. JMP Pro software version 16.0.0 (SAS Institute Japan, Inc.) was used for statistical analysis.

## Funding sources

This work was supported by the Grant-in-Aid for Scientific Research from the Ministry of Education, Culture, Sports, Sciences, and Technology of Japan ( grant number: JP23771889 to A.F.).

## CRediT authorship contribution statement

**Chin Vanessa D:** Writing – review & editing. **Otani Yusuke:** Writing – review & editing. **Peña Tirso:** Writing – review & editing. **Katayama Haruyoshi:** Writing – review & editing, Conceptualization. **Itano Takuto:** Writing – review & editing. **Ando Teruhiko:** Writing – review & editing. **Huang Rongsheng:** Writing – review & editing, Data curation. **Fujimura Atsushi:** Writing – review & editing, Writing – original draft, Visualization, Validation, Supervision, Project administration, Investigation, Funding acquisition, Formal analysis, Data curation, Conceptualization. **Asaka Yutaro:** Visualization, Investigation, Formal analysis, Data curation. **Masumoto Toshio:** Methodology, Investigation, Formal analysis, Data curation. **Uneda Atsuhito:** Methodology, Investigation, Formal analysis, Data curation.

## Declaration of Competing Interest

The authors declare that they have no known competing financial interests or personal relationships that could have appeared to influence the work reported in this paper.

## Data Availability

The data that support the findings of this study are available from the corresponding author upon reasonable request.
